# Whole-Genome Sequencing and Structure Study of Three Biting-Insect–Associated Viruses (*Yunnan Orbivirus*, Guangxi Orbivirus, and Yongshan Totivirus) Isolated in Yunnan, China

**DOI:** 10.1155/av/8321566

**Published:** 2025-08-07

**Authors:** Zhanhong Li, Yingliang Duan, Jianbo Zhu, Le Li

**Affiliations:** ^1^Yunnan Tropical and Subtropical Animal Virus Diseases Laboratory, Yunnan Animal Science and Veterinary Institute, Kunming, China; ^2^Key Laboratory of Transboundary Animal Diseases Prevention and Control (Co-Construction by Ministry and Province), Ministry of Agriculture and Rural Affairs, Kunming, China

## Abstract

Yunnan Province is an area in China with a major prevalence of biting arthropods (including mosquitos, ticks, and *Culicoides*) and arboviruses including dengue virus (DENV), bluetongue virus (BTV), and epizootic hemorrhagic disease virus (EHDV). Therefore, attempts to isolate and detect arboviruses are frequently conducted in Yunnan during the past decades. In this study, a total of three viral strains/isolates (LF6-4, LF3-1, and LF6C2) previously isolated from cattle, goat, and *Culicoides* in Lufeng County, Yunnan Province, between 2022 and 2023 were used for whole-genome sequencing, phylogenetic analysis, and electron microscopic analysis. As a result, these viruses were completely sequenced. Strain LF6-4 isolated from cattle was identified as a putative Serotype 1 Yunnan orbivirus (YUOV). Strain LF3-1 isolated from goats was identified as Guangxi orbivirus (GXOV), and it is the first GXOV strain isolated from this animal. Isolate LF6C2 represented the first totivirus strain isolated from *Culicoides*. The viral particles of all three isolates collected from the infected C6/36 cells were all icosahedral particles with a diameter of approximately 45 nm. However, MDBK cells yielded YUOV and GXOV particles with diameters of approximately 75 nm. This difference may be caused by different viral proliferation/package modes in the different types of host cells.

## 1. Introduction

Yunnan Province, southwestern China, is relatively warm and rich in water sources and forest areas, making it become a perfect spot for the prevalence of arbovirus, and that seriously hazard human and animal health. It is also a popular area for the investigation of transboundary diseases, since it is adjacent to Myanmar, Laos, and Vietnam. Two arboviruses, Yunnan orbivirus (YUOV) and Banna virus (BAV), were first identified in Yunnan [1, 2]. In addition to these two viruses, several arbovirus strains, including but not limited to Akabane virus (AKAV) [3, 4], bluetongue virus (BTV) [5–7], dengue virus (DENV) [8], epizootic hemorrhagic disease virus (EHDV) [9–11], Palyam virus (PALV) [12, 13], Tibet orbivirus (TIBOV) [14, 15], were isolated in Yunnan in the last 2 decades. Therefore, it is imperative to monitor the arbovirus and the insect vector for defending the health of human and animal.

YUOV and Guangxi orbivirus (GXOV) belong to the genus *Orbivirus* of the *Sedoreoviridae* family and possess double-layered capsids and 10 segments of dsRNA [1, 16, 17]. The segments are numbered Seg1 to Seg10 in descending order of the full lengths of the dsRNA segments [1, 16]. The genome encodes a total of seven structural proteins (VP1–VP7) and at least three nonstructural proteins (NS1–NS3) [1, 17, 18]. VP2 and VP7 are the major constituents of the inner capsid and are defined as the T2 and T13 proteins, respectively [1, 17], according to the number of copies in their icosahedral capsid [19, 20]. The T2 and T13 genes of *Orbivirus* are used to classify its viral species [19–21]. A few VP1 proteins are embedded in the inner capsid and function as RNA-dependent RNA polymerase (RdRP) [1, 16]. VP3 and VP5 together construct the outer capsid and are named outer capsid protein 1 (OC1) and OC2, respectively [1, 16, 17]. For *Orbivirus*, OC1 is the crucial protein for classifying serotypes, since it is the ligand of the viral receptor on the cells of mammal hosts and therefore the target of neutralizing antibodies against *Orbivirus* [16, 22, 23].

For *Orbivirus*, there is no clear boundary between the two layers of capsids observed under electron microscope, although they are considered to possess double capsids [16, 24, 25]. The diameters of BTV and TIBOV virions are estimated as 70–75 nm under electron microscope [19, 26], while the diameters of BTV core particles (i.e., viruses without outer capsids) are close to 60 nm [24]. The BTV core particles can be prepared by chymotrypsin treatment in vitro to remove outer capsids and become more infectious to insect cells because BTV infection on insect cells is mediated by the VP7 (T13) protein on the inner capsids [24, 27]. So far, electron microscope photos were taken regularly for *Orbivirus*, but the accurate diameters of orbivirus yielded from various host cells were usually neglected.

The first identified YUOV was isolated from *Culex tritaeniorhynchus* in Yunnan [1], while three other strains of YUOV were isolated from mosquitoes in Indonesia [28], cattle in Japan [29], and white-tailed deer in the United States of America [18]. Therefore, to date, it has been considered a mosquito-borne arbovirus. GXOV was first isolated from cattle in Guangxi Province of China in 2015 and temporarily named after its geographical origin [17]. Subsequently, a few strains isolated from cattle in Japan have been reported [29], but no other GXOV strain has been reported since.

The members of *Totiviridae* have a single dsRNA segment containing two major open reading frames (ORFs), and a single capsid layer [30]. The ORF1 and ORF2 encode capsid protein and RdRP, respectively, and usually have a short overlap between ORF1 and ORF2 [31–33]. These viruses are classified into five genera, namely, *Giardiavirus*, *Leishmaniavirus*, *Totivirus*, *Trichomonasvirus*, and *Victorivirus*, by the International Committee on Taxonomy of Viruses (ICTV) [34]. Their profile of hosts includes fungi, yeast, and protozoan [30, 34]. Recently, several viruses, such as Shanghai totivirus (SHToV) [35], Tianjin totivirus (TJToV) [36], Yongshan totivirus (YSToV), and Yuanmou totivirus (YMToV) [37], have been isolated from mosquitos or bat feces in China and considered as totiviruses, but their hosts are obviously different from that of classical totiviruses [30, 34].

*Culicoides* Latreille (Diptera: Ceratopogonidae) are small biting midges that carry at least 50 viruses [38]. In particular, some species of *Culicoides* are the sole vectors for such *Orbiviruses* as African horse sickness virus (AHSV), BTV, and EHDV [38]. Therefore, *Culicoides* is closely related to epidemics of several animal diseases.

The purpose of this study is to compare the genetic relationship between the viruses (YUOV, GXOV, and YSToV) isolated from Lufeng County and their homogenous strains isolated from both here and abroad and study the structures of these viruses under transmission electron microscope (TEM).

## 2. Materials and Methods

### 2.1. Viral Strains

Viral strains LF6-4 and LF3-1 and isolate LF6C2 were isolated from blood samples and *Culicoides asiana*, respectively, through the *Aedes albopictus* cell line C6/36 in the previous work (Table 1) [39]. These strains were all collected from Lufeng County, Yunnan Province, China.

### 2.2. Viral RNA Preparation

Mosquito cell line C6/36 was used to feed the virus and cultured in minimum essential medium (MEM) with 5% fetal bovine serum (FBS), 100 U/mL penicillin, and 100 μg/mL streptomycin (Gibco, Thermo Fisher Scientific, Grand Island, NY, USA). Media with viral isolates were inoculated to T75 flasks of C6/36 and cultured at 28°C. When cytopathic effects (CPEs) appeared in 90% of cells, the cell pellets were collected after scraping cells and centrifuged at 360 g for 5 min.

Viral genomes were extracted and prepared using methods previously described [11, 15]. Briefly, cell pellets were disintegrated through freezing and thawing twice. Subsequently, the viral RNA was extracted from the cell pellets using an RNAiso-plus kit (Takara) according to the manufacturer's instructions. The RNA was pelleted by using centrifugation (15,000 g for 15 min at 4°C), washed with 1 mL of 75% ethanol, air-dried, and suspended in 50 μL of RNase-free water.

### 2.3. Whole-Genome Sequencing

The viral dsRNA was used to synthesize and amplify the cDNAs of complete genomes using a full-length amplification of cDNA (FLAC) method [11, 40]. The genomic dsRNA of YSToV was acquired successfully but was too long to amplify complete genomic DNA using the FLAC method. Prepared genomic DNA of three viral strains and the genomic dsRNA of YSToV were submitted to the MAGIGENE Company (Guangzhou, China) for sequencing, using a HiSeq 2000 system (Illumina, San Diego, CA, USA) and software SOAPdenovo [41]. For sequencing, a genomic DNA library of YSToV was prepared through reverse transcription (RT) and polymerase chain reaction (PCR) using random primers from the company. Acquired genomic sequences were submitted to GenBank.

### 2.4. Electrophoresis

An 8-μL aliquot of each prepared viral RNA was used for electrophoresis. To clean the cellular DNA and RNA, 1 μL of S1 nuclease (180 U/μL) and 1 μL of S1 nuclease buffer (Takara) were added to each RNA sample, respectively, and the mixtures were incubated at 37°C for 30 min before electrophoresis. The dsRNA fragments were separated using electrophoresis (90 V, 2-3 h) in 1% agarose gel with dye Goldview II (Solarbio, Beijing, China). Fluorescent bands were screened using a Gel Doc XR + machine with Image Lab software (Bio-Rad, Hercules, CA, USA).

### 2.5. Phylogenetic Analysis

All the reference sequences used in this study were downloaded from GenBank and are listed in Tables S1–S3. Complete CDSes containing valid stop codons were used to construct phylogenetic trees, while the incomplete or defective CDSes were not used. For totivirus, the CDS of the RdRP gene started at different sites, which might be caused by the divergence among different researchers in determining the start codon. In this study, the extrusive sequences on the 5′ end of RdRP CDSes from Omono River virus (OMRV) (strains AK4, TB94, TB102, and Y61) and SHToV strain FX17 were truncated.

MEGA 11 software [42] was used to perform sequence alignment and tree construction. Phylogenetic trees were constructed using the Neighbor-Joining (NJ) algorithm (model = Kimura 2, substitution = T + T, gap treatment = pairwise deletion, codon position = 1, bootstrap = 1000) or the Maximum Parsimony (MP) algorithm (type = nucleotide, gap treatment = use all sites, codon position = 1, and bootstrap = 1000) using the CDSes aligned with the MUSCLE (codons) algorithm. The bootstrap values of less than 70% in the trees were omitted.

### 2.6. Electron Microscopic Analysis

A T225 flask of viral isolate infected C6/36 cells showing 90% CPEs at 3 days postinfection (dpi) was frozen and thawed twice to release the virions in the cells, and the supernatant was acquired through centrifugation (1345 g, 30 min) and filtration using a Φ0.22-mm filter. In addition, the same doses of isolates (YUOV/LF6-4 and GXOV/LF3-1) were added into T225 flasks of bovine kidney cell line MDBK, respectively, and the viruses were collected in the same way at 4 dpi (no obvious CPE compared with negative MDBK cells).

The filtrate containing 10% (wt/vol) PEG-8000% and 2% (wt/vol) NaCl was softly stirred with a magnetic rotor (4°C, 80 rpm, overnight). Subsequently, the filtrate was centrifuged (4°C, 7025 g, 1 h), and the deposits were suspended in 5 mL PBS. The suspension was added to 30% (wt/vol) sucrose cushion and then centrifuged (4°C, 222,200 g, 2.5 h). The deposits of viral particles on the bottom were suspended in 200 μL PBS, and a drop was placed onto a copper screen with a Formvar membrane. The sample was stained with 2% phosphotungstic acid for 1.5 min and air-dried. Photographs of viral particles were taken using transmission electron microscopy (AIG2SPIRIT TWIN, Thermo Scientific, USA).

Viral diameters were measured with Photoshop (2020) based on the photos. Median diameters with 95% confidence intervals (CIs) were calculated using PASW Statistics (v18; SPSS Inc., USA).

### 2.7. Quantitative Polymerase Chain Reaction (qPCR) Tests

To compare the YUOV levels in cells C6/36 and MDBK used for the TEM analysis, 50 μL of supernatant from YUOV infected C6/36 (3 dpi) and MDBK (4 dpi), respectively, was subjected to extract viral RNA before cracking the cells, using a MagMAX-96 Viral RNA isolation kit (Ambion, Thermo Fisher Scientific, Waltham, MA, USA) following the manufacturer's directions and a MagMAX Express-24 machine (Ambion, Thermo Fisher Scientific). RNA was eluted with a 50-μL elution buffer for each sample. The two RNA samples and sterile water as negative control were used for qPCR tests. Primers YUOV-qF (5′-GCGTTACAGG AAATTCTTG) and YUOV-qR (5′-GCAGTTTCAA TCTCTTGTC) and probe YUOV-P (5′-FAM-CTGACCACGC AACTGAGACT CT-BHQ1) were used. A 2-μL aliquot of the sample was added to 20 μL of reaction solution prepared using the Quant One Step PrimeScript RT-PCR Kit (Takara) according to the manufacturer's instructions. The RT-qPCR was performed on a Fast7500 realtime PCR machine (Applied Biosystems) at the following cycling conditions: 42°C, 5 min; 95°C, 10 s; 95°C for 5 s, 60°C for 34 s, 40 cycles. Fluorescence was measured at the end of each extension step. The Ct values were shown as mean ± SD (*n* = 3).

## 3. Results

### 3.1. Genomes of Viral Strains

**Figure 1:**
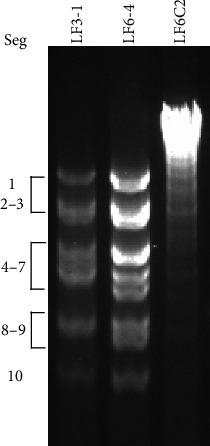
Electrophoresis of the genomes of three viral isolates. The genomic dsRNAs of LF3-1 (GXOV), LF6-4 (YUOV), and LF6C2 (YSToV) were loaded in order.

The genomic RNA of strains LF3-1 (GXOV) and LF6-4 (YUOV) and isolate LF6C2 (YSToV) were separated in agarose gel (Figure 1). The genomes of LF6-4 and LF3-1 each comprised 10 segments and exhibited a similar banding pattern (approximately 3-4-2-1), while the genome of isolate LF6C2 was a single large dsRNA with a length of 7.7 kb.

The sequences of the complete genome of the three isolates were acquired successfully and all stored on GenBank (access number PQ156918- PQ156938). Major data are listed in Tables 2 and 3.

### 3.2. Phylogenetic Analysis

**Figure 2:**
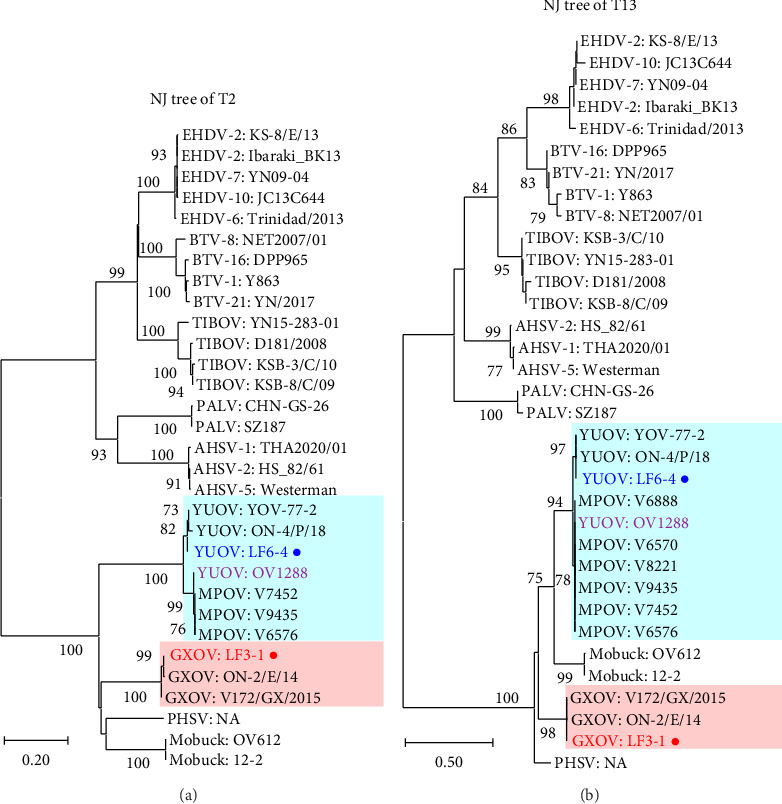
Phylogenetic trees of T2 and T13 for orbivirus. The trees of T2 (a) and T13 (b) were constructed with the NJ algorithm using the complete CDSes of YUOV, GXOV, and other orbiviruses. Viral species and curt strain numbers are shown; our strains are marked by circles. Bootstrap values < 70% omitted.

**Figure 3:**
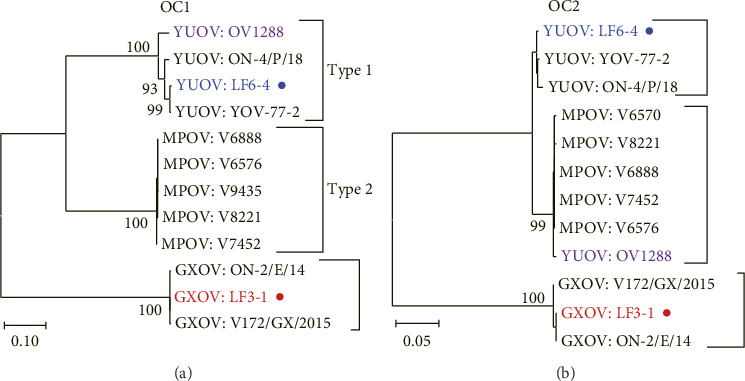
Phylogenetic trees of OC1 and OC2 for three orbiviruses. The trees of the OC1 (= VP3) gene (a) and the OC2 (= VP5) gene (b) were constructed with the NJ algorithm using the complete CDSes from YUOV, GXOV, and MPOV. Viral species and curt strain numbers are shown; our strains are marked by circles. Bootstrap values < 70% omitted.

Our strains LF6-4 and LF3-1 were compared with the reference strains of orbivirus and confirmed as YUOV and GXOV in the phylogenetic trees of the T2, T13 (Figure 2), and VP1 (RdRP) (Figure S1) genes, respectively. GXOVs clustered together, while Middle Point orbiviruses (MPOVs) were located in the cluster of YUOVs. The phylogenetic analysis of OC1 (key gene to determine the serotype of *Orbivirus*) and OC2 suggested that YUOV and MPOV belonged to two putative serotypes of YUOV, while GXOV was different from YUOV and MPOV (Figure 3). YUOV strain OV1288 was placed into the clade of MPOV in the trees of all genes except for VP3/OC1 (Figures 2 and 3 and Figures S1 and S2). The Rioja virus (RIOV), which was marked as YUOV strain Rioja in this study, was found to be the same as YUOV/MPOV based on phylogenetic analysis of the VP6 gene (Figures S2B and S3).

**Figure 4:**
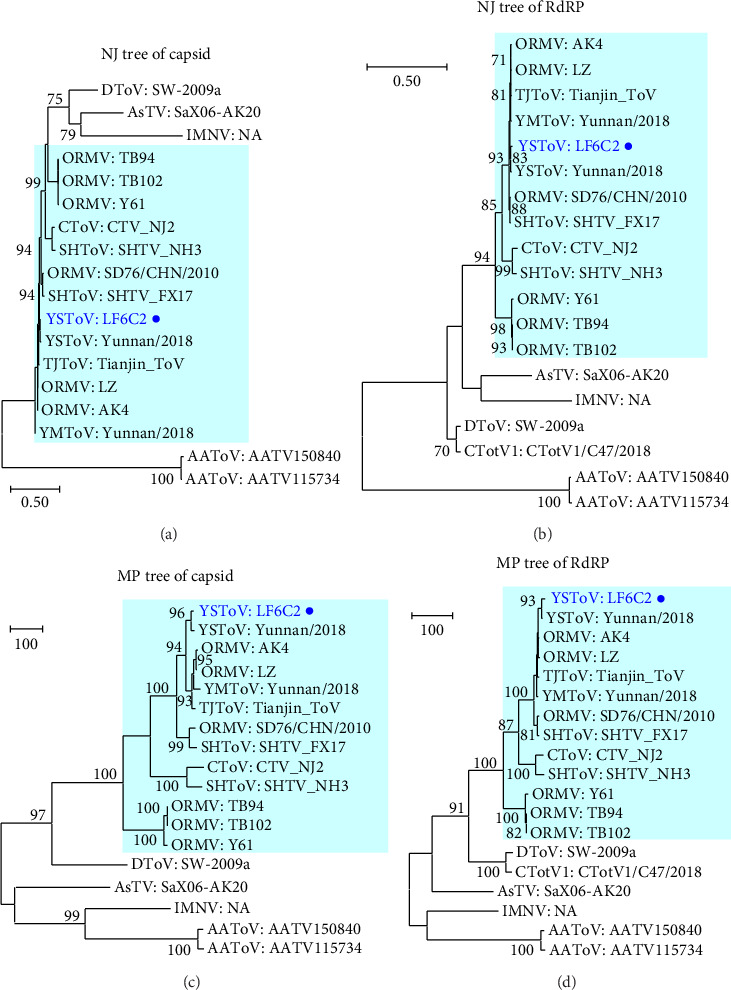
Phylogenetic trees of totivirus. The trees of totivirus genes were constructed with the NJ algorithm using the complete CDS from the totivirus. (a) NJ tree of capsid, (b) NJ tree of RdRP, (c) MP tree of capsid, and (d) MP tree of RdRP. Viral species and curt strain numbers are shown; our strains are marked by circles. Bootstrap values < 70% omitted.

The viral isolate LF6C2 was compared with close totiviruses through the phylogenetic trees of capsid and RdRP, respectively (Figure 4). The patterns of phylogenetic trees of capsid and RdRP were very similar. Isolate LF6C2 was classified as being part of the cluster composed of YSToV, YMToV, SHToV, *Culex* totivirus (CToV), TJToV, and ORMV collected from Asia and was obviously different from the Australian *Anopheles* totivirus (AAToV), penaeid shrimp infectious myonecrosis virus (IMNV), *Armigeres subalbatus* totivirus (AsTV), Drosophila totivirus (DToV), and Clinch totivirus 1 (CTotV1) (Figure 4). Furthermore, isolate LF6C2 was mostly similar to an YSToV (Figure S4). These strains were classified into 4-5 clades, although the genetic distances between any two of them were very short (Figure S4).

### 3.3. Electron Micrograph of Viral Particles

**Figure 5:**
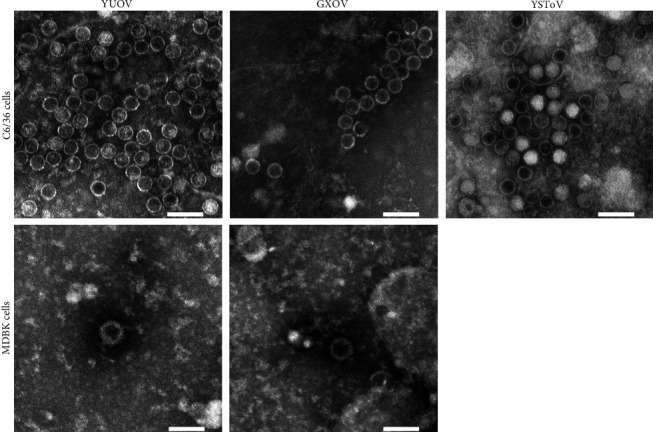
Electron microscope photos of virions for the three viral isolates. The viral particles of the YUOV isolate LF6-4, GXOV isolate LF3-1, and YSToV isolate LF6C2, yielded from C6/36 cells and MDBK cells, were observed under TEM. Scale bar = 100 nm.

The viral particles of the three viral isolates collected from C6/36 cell culture were all nonenveloped and icosahedral under TEM, with similar sizes, and the YUOV and GXOV yielded from MDBK cells were very few and had thicker capsid than that of YUOV and GXOV yielded from C6/36 cells (Figure 5). Virus diameters were measured with Photoshop based on the photos and are shown as median values with a 95% CI in Table 4. The viral particles from C6/36 cell cultures were all approximately 45 nm in diameter, while the viral particles (YUOV/LF6-4 and GXOV/LF3-1) from MDBK cell cultures were approximately 75 nm in diameter (Table 4).

### 3.4. YUOV and GXOV Infection on MDBK

In the experiments for TEM analysis, the MDBK cells infected by YUOV/LF6-4 and GXOV/LF3-1, respectively, did not show discernible CPEs at 4 dpi compared to the normal MDBK cells (negative control) (Figure S5). The supernatant samples of YUOV infected C6/36 and MDBK were collected before TEM experiments and tested by one-step RT-qPCR targeting the YUOV. As a result, the Ct values of C6/36 culture and MDBK culture were 14.42 ± 0.08 and 26.91 ± 0.07, respectively, which means that the MDBK cells are hardly permissive for YUOV yielded from mosquito cells.

## 4. Discussion

*YUOV* was first identified in Yunnan Province, China, and reported in 2005 [1] and has been officially assigned to the genus *Orbivirus* of the *Sedoreoviridae* family by the ICTV [34]. It is evidently different from any other members of the 22 recognized *Orbivirus* in the genomic sequence [26]. To date, YUOV strains have been isolated from mosquitoes, cattle, and deer [1, 28, 29], suggesting that it is a mosquito-borne virus affecting ruminants. Several MPOVs isolated from cattle in Australia were considered to be closely related to YUOV but belonged to another lineage [43, 44], which was confirmed by the phylogenetic analysis in this study. Furthermore, YUOV and MPOV were considered as the putative Serotypes 1 and 2 of YUOV, respectively [45]; therefore, our YUOV strain LF6-4 should belong to the putative YUOV-1. Furthermore, the strain OV1288 isolated from deer in the United States of America was identified as putative Serotype 1 using the phylogenetic analysis of OC1 (VP3) but was located in the clade of MPOV (YUOV-2) in the phylogenetic trees of other genes. This suggested that segment reassortment occurred in this strain. Moreover, six RIOV strains isolated from four mammals (two bovine, one ovine, one dog, and one donkey) with disease and one pool of mosquitoes during an outbreak of Peruvian horse sickness in Peru in 1997 were primarily identified as YUOV strains based on the electropherotype of the genome and the sequences of a few genomic fragments [45]. In this study, the phylogenetic tree of VP6 using the only available complete CDS of RIOV (YUOV strain Rioja) placed the strain Rioja in the YUOV species. If this is the case, the profile of YUOV hosts will be expanded and the YUOV will become a potential risk for livestock. However, the complete CDSes of VP3 from these strains were needed to clarify their serotype or lineage in YUOV species.

GXOV was named for the orbivirus strain isolated from sentinel cattle in Guangxi Province, China, in 2015 [17]. Several GXOV strains were isolated from cattle in Japan [29]. In this study, the phylogenetic analysis confirmed that GXOV was a novel orbivirus species distinguished from other orbiviruses. Our strain LF3-1 was first isolated from goats and expands the range of hosts for GXOV. According to the characteristics of *Orbivirus*, GXOV was considered a potential arbovirus. Thus, more research is needed to establish proof of GXOV infection from biting insects.

Originally, a group of viruses with a single segment of dsRNA containing two ORFs were placed into five genera of *Totiviridae* and specifically parasitized in fungi, yeast, or protozoan [30, 34]. Subsequently, the profile of hosts for tentative totivirus was expanded to shrimp in 2006 [46] fruit flies in 2010 [47], and mosquito in 2010 [33]. Recently, another lineage of tentative totivirus, including CToV, OMRV, SHToV, YMToV, and YSToV, was isolated from mosquitoes [35, 37, 48, 49]. After that, a few YSToV were isolated from *Culicoides* in 2022 [39], which expanded the profile of hosts for totivirus. It is surprising that a Clinch totivirus 1 (CTotV1) sequenced by the metagenomic method with mussel samples was recognized as a totivirus with the ssRNA (+) genome by Richard et al. in 2020 [50]. We used this strain for phylogenetic analysis in this study, although it might have been identified wrongly.

In this study, the viral particles of the three viruses yielded by C6/36 cells were almost the same in shape and size when observed by TEM. For YSToV, the viral shape and size were consistent with those of other totiviruses reported previously (Table 5). However, the diameters (45 nm) of YUOV and GXOV from C6/36 cultures observed in this study were distinctly smaller than other orbiviruses, such as BTV and TIBOV (Table 5) [19, 26]. The diameters of these YUOV and GXOV were close to that of the YUOV observed by Attoui et al. (Table 5), who considered that their YUOV particles lacked outer capsids [1]. Notably, the YUOV and GXOV yielded by MDBK cells had larger diameters (75 nm) which were close to that of intact BTV (Table 5). These suggested that the orbivirus particles yielded from mosquito cells (e.g., C6/36) somehow do not contain the outer layer of the capsid, while mammal hosts will produce virions with double layers of capsids. Accordingly, the outer capsid of *Orbivirus* is useless for infection in insect hosts because orbivirus infection of insect hosts is specifically mediated by the T13 (VP7) protein of the inner capsid following the digestion of the outer capsid occurring in the gut of the insect [16, 27, 55]. However, the MDBK cells seem hardly permissive for YUOV and GXOV yielded from C6/36 cells, although they yielded intact viral particles with thick capsids and diameters of approximately 75 nm. The pool permission of MDBK for YUOV/GXOV may be because the viruses yielded from mosquito cells lack OC1 proteins which mediate the orbivirus invasion to mammal host cells [16, 56, 57]. Alternatively, MDBK cells were just hardly permissive for YUOV/GXOV, no matter the origin of viruses. In *Culicoides*, secondary BTV infection occurs in the salivary glands after initial gut cell infection [58]. Therefore, the orbivirus yielded from insect salivary glands might obtain outer capsids and become infectious to mammal hosts. However, to date, there is no electronographs of BTV, EHDV, and TIBOV collected from mosquito cells and no electronographs of YUOV and GXOV collected from mammal cells (e.g., BHK-21). Further studies are required to compare orbivirus particles produced in mammal cells and mosquito cells. If the two viral proliferation-package modes were common in Orbivirus, it is valuable to study the mechanism of the two viral proliferation-package modes in mammal and insect hosts.

## 5. Conclusions

Three viruses collected from Lufeng County, Yunnan Province, China, in 2022 and 2023 were completely sequenced. Strain LF6C2 was the first totivirus strain isolated from *Culicoides*. Strain LF6-4 isolated from cattle was identified as putative Serotype 1 of YUOV. Strain LF3-1 isolated from goat was identified as GXOV, which is a novel orbivirus described in 2018 and confirmed in this study. This strain is the first GXOV strain isolated from goats. The mosquito host cells (C6/36) are inclined to yield core particles of YUOV and GXOV with diameters of approximately 45 nm, while the mammal host cells (MDBK) yield intact YUOV and GXOV with diameter of approximately 75 nm. However, more detailed studies are needed to clarify the situations and mechanisms of outer capsids packaging for orbivirus in different host cells.

## Figures and Tables

**Table 1:** Sources of viral isolates.

Isolates	Virus	Hosts	Collection dates	Collection sites		
				Latitude (°N)	Longitude (°E)	Elevation (m a.s.l.)
LF6-4	YUOV	Cattle	6 Jul 2022	25.00	101.91	1414
LF6C2	YSToV	*C. asiana*	6 Jul 2022	25.00	101.91	1414
LF3-1	GXOV	Goat	30 Mar 2023	25.30	102.19	1749

**Table 2:** Genomic data of the two strains of orbivirus.

Seg	Gene	LF6-4 (YUOV)			LF3-1 (GXOV)		
		Length (bp)	CDS range	Accession number	Length (bp)	CDS range	Accession number
1	VP1 (RdRP)	3993	14–3961	PQ156928	3996	14–3964	PQ156918
2	VP2 (T2)	2900	12–2834	PQ156929	2914	18–2831	PQ156919
3	VP3 (OC1)	2688	19–2640	PQ156930	2680	18–2630	PQ156920
4	VP4	1993	8–1945	PQ156931	2000	8–1951	PQ156921
5	NS1	1958	31–1728	PQ156932	1847	31–1725	PQ156922
6	VP5 (OC2)	1683	42–1649	PQ156933	1687	39–1637	PQ156923
7	NS2	1506	30–1337	PQ156934	1620	52–1359	PQ156924
8	VP7 (T13)	1191	20–1087	PQ156935	1182	20–1081	PQ156925
9	VP6	1082	18–1034	PQ156936	1134	17–1087	PQ156926
10	NS3	825	16–777	PQ156937	829	16–783	PQ156927

**Table 3:** Genomic data of YSToV isolate LF6C2.

Genome/gene	Gene/protein	Length or CDS range (bp)	Access number
Genome	NA	7706	PQ156938
ORF1	Capsid	74–5131	/
ORF2	RdRP	5386–7602	/

**Table 4:** Diameters of the three isolates.

Isolate	Virus	Cells	Diameter, medial (95% CI range) (nm)	Counted number
LF6-4	YUOV	C6/36	44.7 (43.8, 45.5)	60
		MDBK	75.6 (72.4, 82.1)	3

LF3-1	GXOV	C6/36	44.4 (43.5, 45.2)	20
		MDBK	73.5 (71.4, 75.8)	4

LF6C2	YSToV	C6/36	45.2 (44.3, 46.2)	46

**Table 5:** Comparison of the particle diameters from associated viruses.

Virus	Cells	Sample type	Diameter of viral particle (nm)	Length of genome (kb)	Reference
*Orbivirus*					
BTV	BHK-21	Viral particles	70 (virion/ISVP)^a^ 58 (core particle)	19.2	[19, 24, 25, 51]
PHSV^b^	C6/36	Infected cells	60	19.7	Attoui et al. [45]
TIBOV	BHK-21	Viral particles	71–75	19.2	[26, 52]
YUOV	C6/36	Viral particles	≈ 50 (core particle)	19.8	Attoui et al. [1]

*Totivirus*					
AsTV	C6/36	Viral particles	≈ 40	7.5	Zhai et al. [33]
CToV	C6/36	Infected cells, viral particles	≈ 40	7.6	Li et al. [53]
OMRV	C6/36	Infected cells, viral particles	≈ 40	7.6	Isawa et al. [48]
TJToV	Insect cell	Viral particles	40–43	7.6	Yang et al. [36]
TkTV1^c^	Fungi	Viral particles	38–40	4.7	Khalifa et al. [54]

^a^ISVP: Infectious subviral particle.

^b^PHSV: Peruvian horse sickness virus.

^c^TkTV1: Trichoderma koningiopsis totivirus 1.

## Data Availability

All data and materials are fully available as supporting information, and sequencing information can be accessed on NCBI (https://www.ncbi.nlm.nih.gov) through accession numbers PQ156918-PQ156938.

## Supporting Information

Supporting Information 1 Table S1: Collected data of reference viral strains.

Supporting Information 2 Table S2: Viral sequences of the orbiviruses downloaded from GenBank and used in this study.

Supporting Information 3 Table S3: Viral genomes of the totiviruses downloaded from GenBank and used in this study.

Supporting Information 4 Figure S1: Phylogenetic trees of the VP1 gene for *Orbivirus*.

Supporting Information 5 Figure S2: Phylogenetic trees of five genes from three orbiviruses.

Supporting Information 6 Figure S3: Phylogenetic trees for classifying strain Rioja.

Supporting Information 7 Figure S4: Phylogenetic trees of YSToV and close totivirus.

Supporting Information 8 Figure S5: Photos of MDBK cells.
