# Supplementary material for: Whole-Genome Sequencing and Structure Study of Three Biting-Insect–Associated Viruses (Yunnan Orbivirus, Guangxi Orbivirus, and Yongshan Totivirus) Isolated in Yunnan, China
Source: Adv Virol. 2025 Aug 7;2025:8321566. doi: 10.1155/av/8321566 (PMC12352999; doi:10.1155/av/8321566)
Supplement: Supporting Information 1 — Table S1: Collected data of reference viral strains. [file 8321566.f1.docx]

TABLE S1: Collected data of reference viral strains.

| Viral strain | |  | Source of virus | | |
| --- | --- | --- | --- | --- | --- |
| Species | Full ID number |  | year | Country | Host |
| **Orbivirus** |  |  |  |  |  |
| AHSV-1 | THA2020/01 |  | 2020 | Thailand | Horse |
| AHSV-2 | HS_82/61 |  | 1961 | South Africa | Horse |
| AHSV-5 | Westerman |  | 1936 | South Africa | Horse |
| BTV-1 | Y863 |  | 1979 | China | Sheep |
| BTV-8 | NET2007/01 |  | 2007 | Netherlands | Bovine |
| BTV-16 | DPP965 |  | 1987 | Australia | Cattle |
| BTV-21 | YN/2017 |  | 2017 | China | Goat |
| EHDV-2 | KS-8/E/13 |  | 2013 | Japan | Cattle |
| EHDV-6 | Trinidad/EHDV-6/2013 |  | 2013 | Trinidad and Tobago | Bovine |
| EHDV-7 | YN09-04 |  | 2013 | China | Cattle |
| EHDV-10 | JC13C644 |  | 2013 | China | *Culicoides* |
| EHDV-2 | BK13 |  | 1997 | Japan | Cattle |
| PALV | CHN-GS-26 |  | 2016 | China | Yak |
| PALV | SZ187 |  | 2012 | China | Cattle |
| TIBOV | D181/2008 |  | 2008 | China | Mosquito |
| TIBOV | KSB-3/C/10 |  | 2010 | Japan | *Culicoides sp.* |
| TIBOV | KSB-8/C/09 |  | 2009 | Japan | *Culicoides sp.* |
| TIBOV | YN15-283-01 |  | 2015 | China | *Culicoides sp.* |
| YUOV | YOV-77-2 |  | ≤ 2005 | China | *Culex tritaeniorhynchus* |
| YUOV | OV1288 |  | 2019 | USA | white-tailed deer |
| YUOV | ON-4/P/18 |  | 2018 | Japan | Cattle |
| YUOV | Rioja |  | 1997 | Peru | Bovine, ovine, dog, donkey, or mosquito ^a)^ |
| MPOV | MPOV-V6570 |  | 2005 | Australia | Cattle |
| MPOV | MPOV-V6888 |  | 2006 | Australia | Cattle |
| MPOV | MPOV-V8221 |  | 2010 | Australia | Cattle |
| MPOV | MPOV-V9435 |  | 2016 | Australia | Cattle |
| MPOV | MPOV-V7452 |  | 2008 | Australia | Cattle |
| MPOV | MPOV-V6576 |  | 2005 | Australia | Cattle |
| GXOV | V172/GX/2015 |  | 2015 | China | Cattle |
| GXOV | ON-2/E/14 |  | 2014 | Japan | Cattle |
| **Totivirus** |  |  |  |  |  |
| AAToV ^b)^ | AATV 150840 |  | 2005 | Australia | *Anopheles hinesorum* |
| AAToV | AATV 115734 |  | 2007 | Australia | *Anopheles annulipes* |
| AsTV ^b)^ | SaX06-AK20 |  | 2006 | China | *Armigeres subalbatus* |
| CTotV1 ^b)^ | CTotV1/C47/2018 |  | 2018 | USA | *Actinonaias pectorosa* |
| CToV ^b)^ | CTV_NJ2 |  | 2010 | China | *Culex tritaeniorhynchus* |
| DToV ^b)^ | SW-2009a |  | 2009 | USA | *Drosophila melanogaster* |
| IMNV ^b)^ | NA |  | NA | Brazil | *Penaeus vannamei* |
| OMRV | AK4 |  | 2005 | Japan | *Culex pipiens pallens* |
| OMRV | LZ |  | 2017 | China | *Aedes albopictus* |
| OMRV | SD76/CHN/2010 |  | 2010 | China | *Culex tritaeniorhynchus* |
| OMRV | TB94 |  | 2011 | Vietnam | *Culex vishnui* |
| OMRV | TB102 |  | 2011 | Vietnam | *Culex vishnui* |
| OMRV | Y61 |  | 2007 | Japan | *Culex inatomii* |
| SHToV | SHTV_FX17 |  | 2007 | China | *Culex tritaeniorhynchus* |
| SHToV | SHTV_NH3 |  | 2007 | China | *Culex tritaeniorhynchus* |
| TJToV | Tianjin |  | 2007 | China | From bat feces |
| YSToV | Yunnan/2018 |  | 2012 | China | *Culex tritaeniorhynchus* |
| YMToV | Yunnan/2018 |  | 2018 | China | *Culex tritaeniorhynchus* |

a) Six viral isolates were isolated from 5 animals, and one of isolates was sequenced.

b) AAToV = Australian *Anopheles* totivirus; AsTV = *Armigeres subalbatus* totivirus; CTotV1 = Clinch totivirus 1 (it was described as ssRNA virus by Richard *et al*, 2020); CToV= *Culex tritaeniorhynchus* totivirus; DToV = *Drosophila* totivirus; IMNV = Penaeid shrimp infectious myonecrosis virus.
