# Supplementary material for: Whole-Genome Sequencing and Structure Study of Three Biting-Insect–Associated Viruses (Yunnan Orbivirus, Guangxi Orbivirus, and Yongshan Totivirus) Isolated in Yunnan, China
Source: Adv Virol. 2025 Aug 7;2025:8321566. doi: 10.1155/av/8321566 (PMC12352999; doi:10.1155/av/8321566)
Supplement: Supporting Information 2 — Table S2: Viral sequences of the orbiviruses downloaded from GenBank and used in this study. [file 8321566.f2.docx]

TABLE S2: Viral sequences of the orbiviruses downloaded from GenBank and used in this study.

|  |  |  |  |  |  |  |  |  |  |  |
| --- | --- | --- | --- | --- | --- | --- | --- | --- | --- | --- |
| Strain ID | VP1/RdRP | T2 | T13 | OC1 | OC2/VP5 | VP4 | VP6 | NS1 | NS2 | NS3 |
| **AHSV** |  |  |  |  |  |  |  |  |  |  |
| THA2020/01 | MT586213.1 | MT586214.1 | MT586218.1 | NA | MT586217.1 | MT586215.1 | MT586220.1 | MT586216.1 | MT586219.1 | MT586221.1 |
| HS_82/61 | KF859996.1 | KF859998.1 | KF860002.1 | KF859997.1 | KF860001.1 | KF859999.1 | KF860003.1 | KF860000.1 | KF860004.1 | KF860005.1 |
| Westerman | KP009781.1 | KP009783.1 | KP009787.1 | KP009782.1 | KP009786.1 | KP009784.1 | KP009789.1 | KP009785.1 | KP009788.1 | KP009790.1 |
| **BTV** |  |  |  |  |  |  |  |  |  |  |
| Y863 | KC879615.1 | KC879617.1 | KC879621.1 | KC879616.1 | KC879620.1 | KC879618.1 | KC879623.1 | KC879619.1 | KC879622.1 | KC879624.1 |
| NET2007/01 | GQ506451.1 | GQ506453.1 | GQ506457.1 | GQ506452.1 | GQ506456.1 | GQ506454.1 | GQ506459.1 | GQ506455.1 | GQ506458.1 | GQ506460.1 |
| DPP965 | JQ086231.1 | JQ086233.1 | JQ086237.1 | JQ086232.1 | JQ086236.1 | JQ086234.1 | JQ086239.1 | JQ086235.1 | JQ086238.1 | JQ086240.1 |
| YN/2017 | MK250956.1 | MK250958.1 | MK250962.1 | MK250957.1 | MK250961.1 | MK250959.1 | MK250964.1 | MK250960.1 | MK250963.1 | MK250965.1 |
| **EHDV** |  |  |  |  |  |  |  |  |  |  |
| KS-8/E/13 | LC599911.1 | LC202974.1 | LC599914.1 | LC202952.1 | LC202963.1 | LC599912.1 | LC599916.1 | LC599913.1 | LC599915.1 | LC599917.1 |
| BK13 | KM509050.1 | KM509052.1 | KM509056.1 | KM509051.1 | KM509055.1 | KM509053.1 | KM509058.1 | KM509054.1 | KM509057.1 | KM509059.1 |
| Trinidad/2013 | MK919254.1 | MK919256.1 | MK919260.1 | MK919255.1 | MK919259.1 | MK919257.1 | MK919262.1 | MK919258.1 | MK919261.1 | MK919263.1 |
| YN09-04 | MK656453.1 | MK656455.1 | MK656459.1 | MK656454.1 | MK656458.1 | MK656456.1 | MK656461.1 | MK656457.1 | MK656460.1 | MK656462.1 |
| JC13C644 | MT013324.1 | MT013326.1 | MT013330.1 | MT013325.1 | MT013329.1 | MT013327.1 | MT013332.1 | MT013328.1 | MT013331.1 | MT013333.1 |
| **PALV** |  |  |  |  |  |  |  |  |  |  |
| CHN-GS-26 | MH090057.1 | MH090060.1 | MH090069.1 | MF642327.1 | MF642330.1 | MH090063.1 | MH090075.1 | MH090066.1 | MH090072.1 | MH090078.1 |
| SZ187 | KT002588.1 | KT002590.1 | KT002594.1 | KT002589.1 | KT002593.1 | KT002591.1 | KT002596.1 | KT002592.1 | KT002595.1 | KT002597.1 |
| **TIBOV** |  |  |  |  |  |  |  |  |  |  |
| D181/2008 | KR822286.1 | KR822288.1 | KR822292.1 | KR822287.2 | KR822291.1 | KR822289.1 | KR822294.1 | KR822290.1 | KR822293.1 | KR822295.1 |
| KSB-3/C/10 | LC567102.1 | LC567104.1 | LC567108.1 | LC567103.1 | LC567107.1 | LC567105.1 | LC567110.1 | LC567106.1 | LC567109.1 | LC567111.1 |
| KSB-8/C/09 | LC567112.1 | LC567114.1 | LC567118.1 | LC567113.1 | LC567117.1 | LC567115.1 | LC567120.1 | LC567116.1 | LC567119.1 | LC567121.1 |
| YN15-283-01 | MT793636.1 | MT793638.1 | MT793642.1 | MT793637.1 | MT793641.1 | MT793639.1 | MT793644.1 | MT793640.1 | MT793643.1 | MT793645.1 |
| **YUOV** |  |  |  |  |  |  |  |  |  |  |
| YOV-77-2 | AY701509.1 | AY701510.1 | AY701516.1 | AY701511.1 | AY701514.1 | AY701512.1 | AY701517.1 | Abnormal | AY701515.1 | AY701518.1 |
| OV1288 | MW424401.1 | MW424402.1 | MW424407.1 | MW424403.1 | MW424405.1 | MW424404.1 | MW424406.1 | MW424408.1 | MW424409.1 | MW424410.1 |
| ON-4/P/18 | LC585872.1 | LC585873.1 | LC585879.1 | LC585874.1 | LC585877.1 | LC585875.1 | LC585880.1 | LC585876.1 | LC585878.1 | LC585881.1 |
| Rioja | NA | NA | NA | NA | NA | NA | FJ225402.1 | NA | NA | NA |
| **GXOV** |  |  |  |  |  |  |  |  |  |  |
| V172/GX/2015 | NC_040478.1 | NC_040479.1 | NC_040477.1 | NC_040476.1 | NC_040480.1 | NC_040847.1 | NC_040482.1 | NC_040481.1 | NC_040474.1 | NC_040475.1 |
| ON-2/E/14 | LC585908.1 | LC585909.1 | LC585915.1 | LC585910.1 | LC585913.1 | LC585911.1 | LC585916.1 | LC585912.1 | LC585914.1 | LC585917.1 |
| **MPOV** |  |  |  |  |  |  |  |  |  |  |
| V6570 | Incomplete | Incomplete | MZ079682.1 | Incomplete | MZ079680.1 | Incomplete | Incomplete | Incomplete | MZ079681.1 | MZ079684.1 |
| V6888 | MZ079695.1 | Incomplete | MZ079702.1 | MZ079697.1 | MZ079700.1 | Incomplete | MZ079703.1 | MZ079699.1 | MZ079701.1 | MZ079704.1 |
| V8221 | Incomplete | Incomplete | MZ079752.1 | MZ079747.1 | MZ079750.1 | Incomplete | Incomplete | MZ079749.1 | MZ079751.1 | MZ079754.1 |
| V9435 | Incomplete | MZ079816.1 | MZ079822.1 | MZ079817.1 | Incomplete | Incomplete | Incomplete | MZ079819.1 | MZ079821.1 | MZ079824.1 |
| V7452 | MZ079715.1 | MZ079716.1 | MZ079722.1 | MZ079717.1 | MZ079720.1 | MZ079718.1 | MZ079723.1 | MZ079719.1 | MZ079721.1 | MZ079724.1 |
| V6576 | Incomplete | MZ079686.1 | MZ079692.1 | MZ079687.1 | MZ079690.1 | Incomplete | MZ079693.1 | MZ079689.1 | MZ079691.1 | MZ079694.1 |

Note: NA, not available; abnormal and incomplete CDS were not used in this study.
