# Supplementary material for: Whole-Genome Sequencing and Structure Study of Three Biting-Insect–Associated Viruses (Yunnan Orbivirus, Guangxi Orbivirus, and Yongshan Totivirus) Isolated in Yunnan, China
Source: Adv Virol. 2025 Aug 7;2025:8321566. doi: 10.1155/av/8321566 (PMC12352999; doi:10.1155/av/8321566)
Supplement: Supporting Information 3 — Table S3: Viral genomes of the totiviruses downloaded from GenBank and used in this study. [file 8321566.f3.docx]

TABLE S3: Viral genomes of the totiviruses downloaded from GenBank and used in this study.

| Virus | Strain ID | Access number |
| --- | --- | --- |
| Australian *Anopheles* totivirus (AATV) | AATV_150840 | NC_035674.1 |
|  | AATV_115734 | MF073201.1 |
| *Armigeres subalbatus* totivirus (AsTV) | SaX06-AK20 | EU715328.1 |
| Clinch totivirus 1 (CTotV1) * | CTotV1/C47/2018 | MT341489.1 |
| *Culex tritaeniorhynchus* totivirus (CToV) | CTV_NJ2 | KX456218.1 |
| *Drosophila* totivirus (DToV) | SW-2009a | NC_013499.1 |
| Omono River virus (OMRV) | AK4 | AB555544.1 |
|  | LZ | MT066059.1 |
|  | SD76/CHN/2010 | ON402520.1 |
|  | TB94 | KY264025.1 |
|  | TB102 | KY264024.1 |
|  | Y61 | AB555545.1 |
| Penaeid shrimp infectious myonecrosis virus (IMNV) | NA | AY570982.3 |
| Shanghai totivirus (SHToV) | SHTV_FX17 | MN196675.1 |
|  | SHTV_NH3 | MN196674.1 |
| Tianjin totivirus (TJToV) | Tianjin | NC_017084.1 |
| Yongshan totivirus (YSToV) | Yunnan/2018 | MN176215.1 |
| Yuanmou totivirus (YMToV) | Yunnan/2018 | MN176214.1 |

* RdRP CDS only.
