# Supplementary figures and images for: Whole-Genome Sequencing and Structure Study of Three Biting-Insect–Associated Viruses (Yunnan Orbivirus, Guangxi Orbivirus, and Yongshan Totivirus) Isolated in Yunnan, China
Source: Adv Virol. 2025 Aug 7;2025:8321566. doi: 10.1155/av/8321566 (PMC12352999; doi:10.1155/av/8321566)

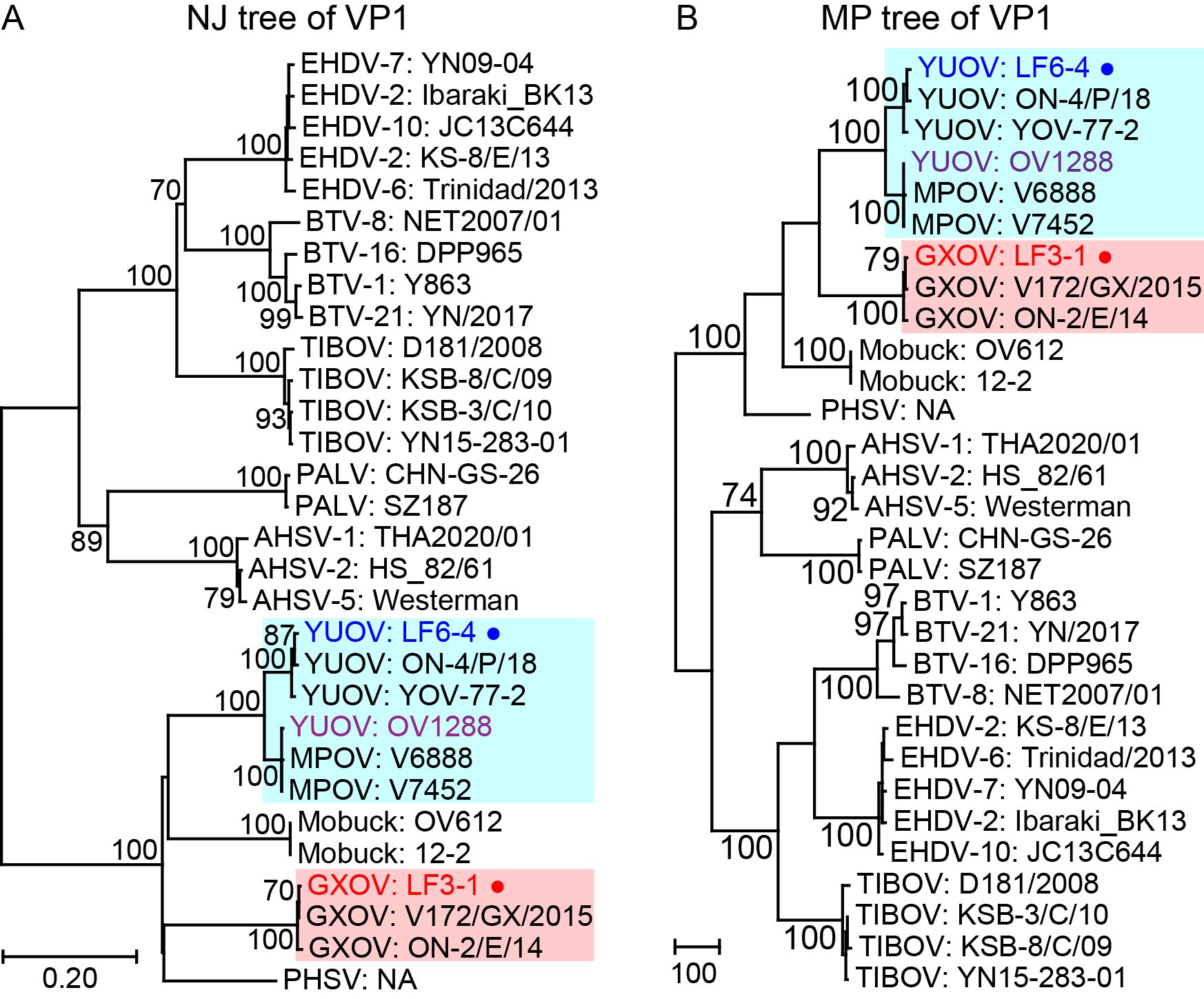

Supplement: Supporting Information 4 — Figure S1: Phylogenetic trees of the VP1 gene for Orbivirus. [file 8321566.f4.jpg]

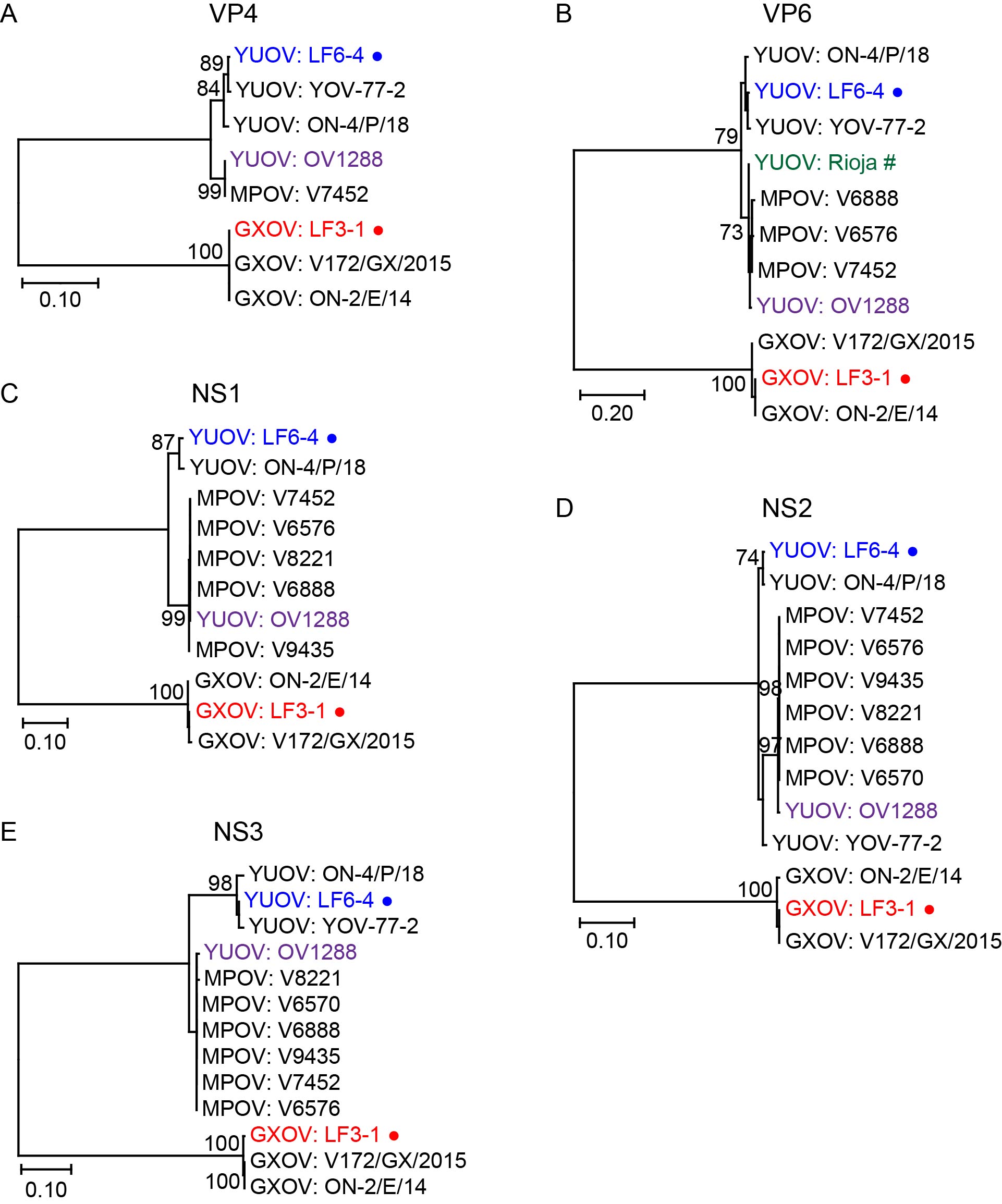

Supplement: Supporting Information 5 — Figure S2: Phylogenetic trees of five genes from three orbiviruses. [file 8321566.f5.jpg]

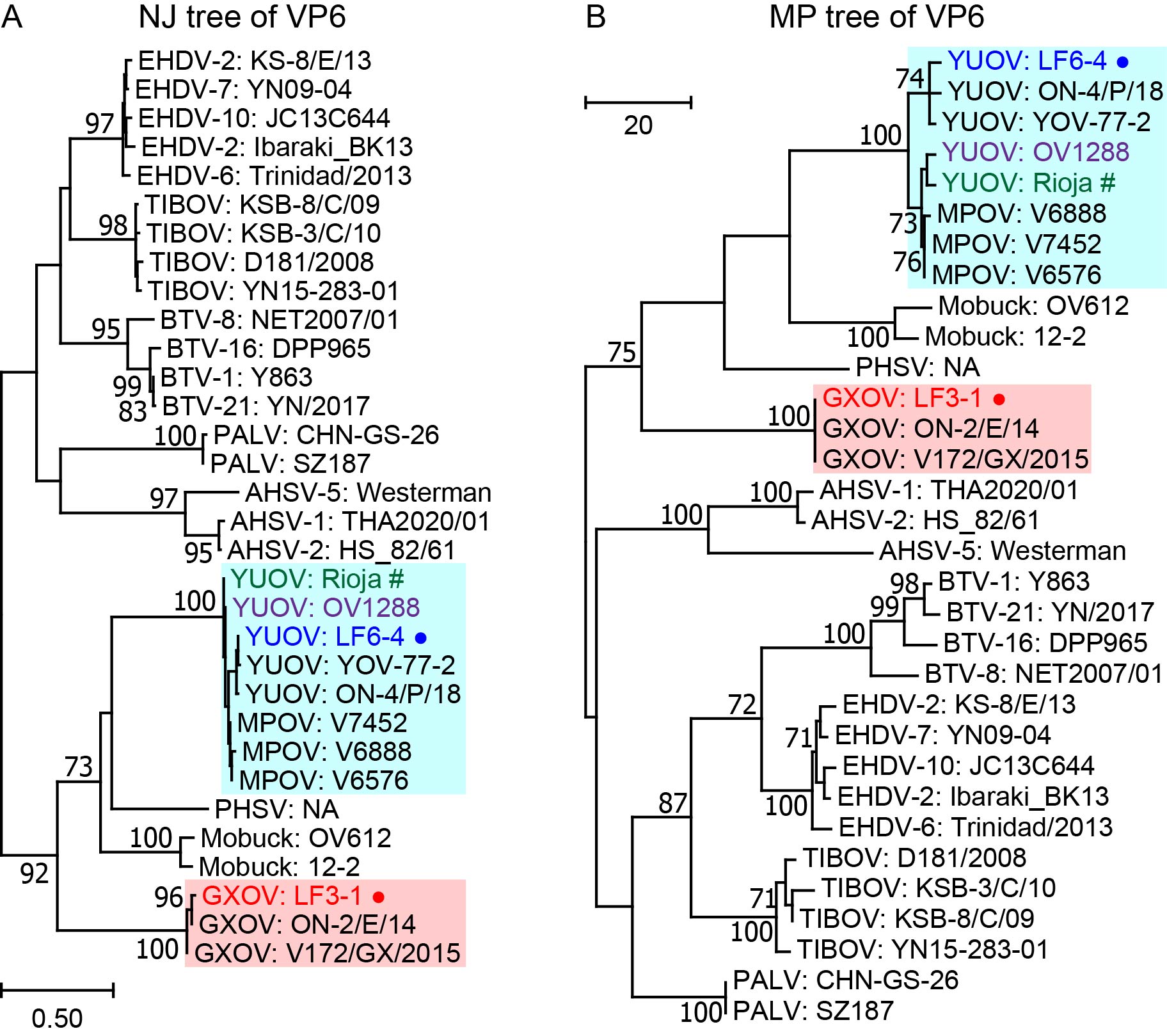

Supplement: Supporting Information 6 — Figure S3: Phylogenetic trees for classifying strain Rioja. [file 8321566.f6.jpg]

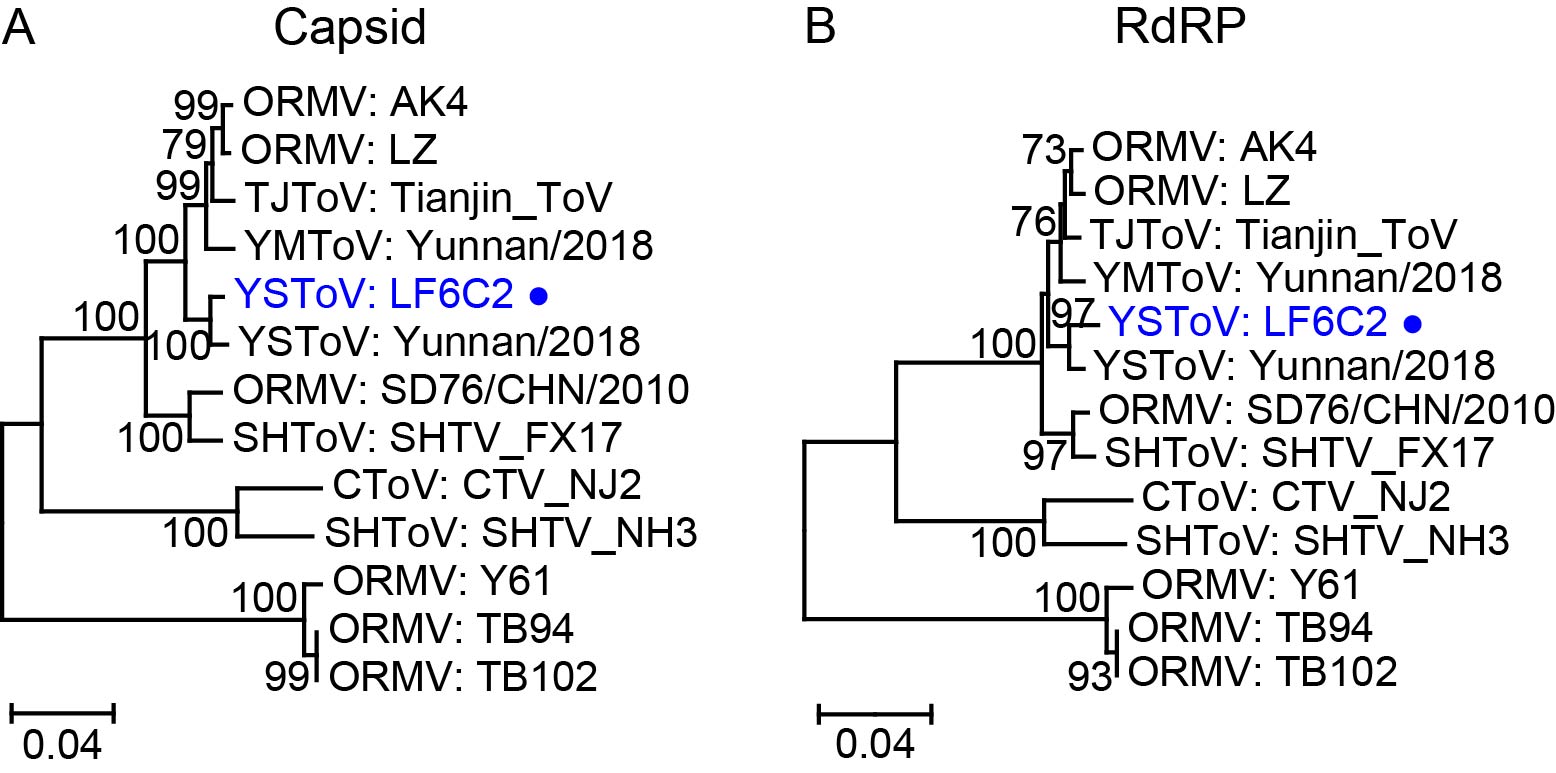

Supplement: Supporting Information 7 — Figure S4: Phylogenetic trees of YSToV and close totivirus. [file 8321566.f7.jpg]

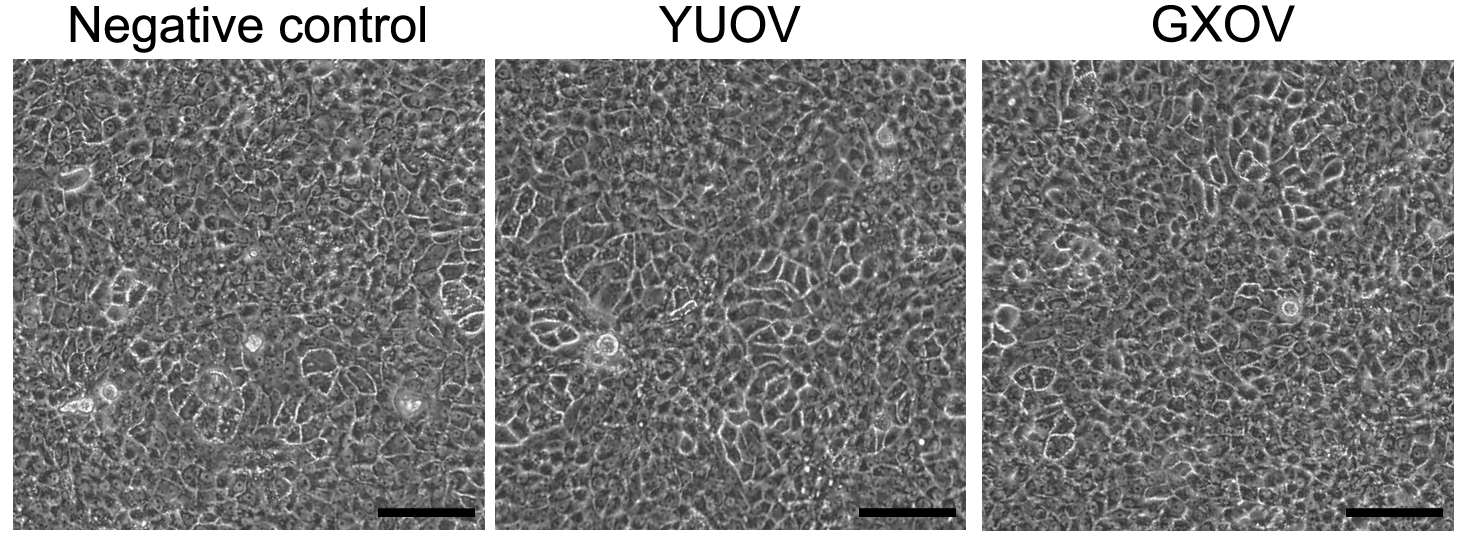

Supplement: Supporting Information 8 — Figure S5: Photos of MDBK cells. [file 8321566.f8.tif]
